# T-cell receptor Vβ repertoire skewing reflects premature immune senescence in children with chronic kidney disease

**DOI:** 10.1007/s00467-026-07267-w

**Published:** 2026-03-25

**Authors:** Emine Ülgen, Seha Saygılı, Ayça Kıykım, Nihan Burtecene, Esra Karabağ Yılmaz, Ayşe Ağbaş, Nur Canpolat

**Affiliations:** 1 Department of Pediatric Immunology and Allergy, Ege University Faculty of Medicine, İzmir, Türkiye; 2 Department of Pediatric Nephrology, Cerrahpaşa Faculty of Medicine, Istanbul University-Cerrahpaşa, Istanbul, Türkiye; 3 Department of Pediatric Immunology and Allergy, Cerrahpaşa Faculty of Medicine, Istanbul University-Cerrahpaşa, Istanbul, Türkiye

**Keywords:** Children, Chronic kidney disease, Premature immune aging, Immune senescence, T-cell receptor repertoire

## Abstract

**Background:**

Chronic kidney disease (CKD), characterized by chronic inflammation and uremic toxicity, represents a state of premature immune aging. However, data on T-cell receptor (TCR) repertoire alterations in pediatric CKD are limited. The TCR variable beta (Vβ) region defines distinct T-cell subfamilies generated by V(D)J recombination and reflects T-cell repertoire diversity and clonal composition. Alterations in TCR Vβ distribution indicate repertoire remodeling associated with chronic antigenic stimulation and immune aging beyond quantitative lymphocyte changes. This study aimed to characterize TCR Vβ family repertoire in children with advanced CKD and explore their associations with clinical and biochemical parameters.

**Methods:**

In this single-center, cross-sectional study, 35 children with CKD stages 3b–5 (21 non-dialysis, 14 dialysis) and 15 age- and sex-matched healthy controls were enrolled. Peripheral blood lymphocyte subsets and TCR Vβ1–Vβ23 distributions were assessed by flow cytometry and compared with clinical and laboratory measures. Group comparisons were performed using the Mann–Whitney *U* test, and associations were assessed using Spearman correlation analysis.

**Results:**

Children with CKD exhibited a skewed TCR Vβ repertoire, with reduced expression of Vβ9 and Vβ11 and increased Vβ17 (*p* = 0.041, 0.001, 0.014, respectively) with corresponding moderate-to-large effect sizes (*r* = 0.37, *r* = 0.61, and *r* = 0.44). Dialysis patients showed lower Vβ11 and higher Vβ12 expression compared with non-dialysis patients (*p* = 0.034 and *p* = 0.048), with large effect sizes (*r* = 0.68 and *r* = 0.66). Reduced Vβ9 correlated with low BMI and higher proteinuria, and reduced Vβ11 correlated with hypoalbuminemia, whereas elevated Vβ12 was associated with higher CRP and creatinine levels. Total lymphocyte counts were preserved, although dialysis patients demonstrated a higher CD4/CD8 ratio.

**Conclusions:**

Pediatric CKD is associated with selective and non-uniform remodeling of the T-cell repertoire, reflecting premature immune senescence. The associations between TCR Vβ alterations, inflammation, and nutritional markers suggest synergistic effects of uremic toxicity and protein-energy imbalance on immune aging, warranting larger mechanistic studies.

**Graphical Abstract:**

A higher resolution version of the  Graphical abstract is available as Supplementary information.

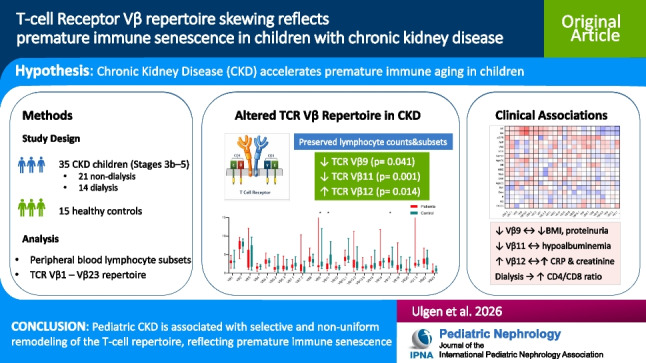

**Supplementary Information:**

The online version contains supplementary material available at 10.1007/s00467-026-07267-w.

## Introduction

Chronic kidney disease (CKD) is increasingly recognized as a state of premature immune aging and dysfunction. Progressive loss of kidney function creates a milieu of uremia, oxidative stress, and chronic inflammation, which disrupts both innate and adaptive immunity [1–3]. These alterations contribute to increased susceptibility to infections, reduced vaccine responses, enhanced risk of virus-related malignancies, and accelerated atherosclerosis [4–7].

Immune dysregulation in CKD encompasses multiple mechanisms, including defective phagocytosis, impaired antigen presentation, reduced antibody production, and impaired T-cell–mediated immunity [8, 9]. Among these, T-cell alterations such as reduced naive T-cell counts, repertoire restriction (oligoclonality), telomere shortening, and accumulation of senescent phenotypes are particularly suggestive of immune senescence [10–12]. The T-cell receptor (TCR), composed of α/β or γ/δ chains, mediates antigen recognition through a diverse process of V(D)J recombination. The β (Vβ) chain is a critical determinant of repertoire diversity and clonal distribution, reflecting the specificity and adaptability of the T-cell–mediated immune response [13, 14].

TCR Vβ skewing has been described as a hallmark of immune senescence in adult CKD subjects [15, 16]; however, data on TCR repertoire changes in the pediatric CKD population are still scarce. Given that immune maturation and aging follow distinct trajectories during childhood, findings from adults cannot be directly applied to children. We hypothesized that children and adolescents with advanced CKD exhibit early, senescence-like alterations in the TCR Vβ repertoire. Therefore, this study aimed to characterize TCR Vβ family distributions in pediatric CKD and to explore their associations with clinical and biochemical parameters.

## Methods

### Study population

**Fig. 1:**
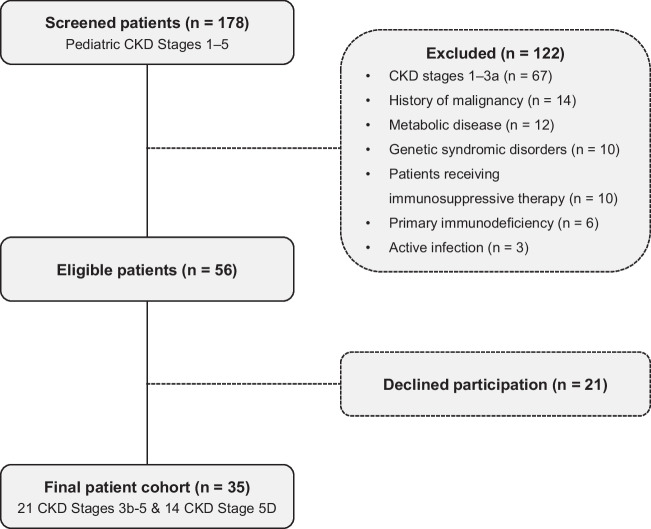
The flowchart illustrates the selection process of the patient population. *CKD* chronic kidney disease, *5D* 5 dialysis

This single-center, observational, cross-sectional case–control study was conducted at a tertiary pediatric nephrology center. A total of 178 patients with a diagnosis of CKD between 2020 and 2021 were screened. The inclusion criteria were an age between 2 and 21 years at the time of enrollment and a CKD stage of 3b or higher (glomerular filtration rate, GFR < 45 mL/min/1.73 m^2^). Patients with primary immunodeficiency, active infection, history of malignancy, metabolic disease-related CKD, or cases receiving immunosuppressive therapy were excluded. After applying these criteria, 35 eligible patients were enrolled in the study, comprising 21 non-dialysis and 14 dialysis patients (Fig. 1).

The control group consisted of 15 age- and sex-matched healthy children who volunteered to participate in the study.

The study protocol was approved by the Institutional Ethics Committee of Istanbul University–Cerrahpaşa (Approval No: 72378, Date: July 7, 2020). Written informed consent was obtained from all participants or their legal guardians.

### Clinical and demographic data

Demographic and clinical data included age, sex, height, weight, body mass index (BMI), height *z*-score, BMI *z*-score (adjusted for height age), as well as primary kidney disease, disease duration, medications, and kidney replacement therapy (KRT). The estimated glomerular filtration rate (eGFR) was calculated using serum creatinine measured by standard biochemical methods and estimated using the CKiD U25 equation in accordance with contemporary pediatric recommendations [17].

### Laboratory tests

Laboratory evaluations included a complete blood count (CBC), serum biochemistry (urea, creatinine, albumin, CRP, calcium, phosphate, alkaline phosphatase (ALP), vitamin D, parathyroid hormone (PTH), immunoglobulin levels (IgG, IgA, IgM), urinalysis, and assessment of proteinuria and microalbuminuria.

### Immunophenotyping and TCR Vβ repertoire analysis

Peripheral blood samples obtained simultaneously with the CBC were analyzed by flow cytometry to determine the distribution of CD3+, CD4+, CD8+ T lymphocytes, CD19+ B lymphocytes, CD16/56+ NK cells, and TCR α/β and γ/δ T-cell subsets. Reference ranges were based on values established for healthy Turkish children [18]. Briefly, whole blood was incubated with monoclonal antibodies against surface markers at room temperature for 20 min. After red cell lysis, cells were washed and proceeded with flow acquisition. The TCR Vβ repertoire was evaluated using flow cytometry with monoclonal antibodies specific to CD3+ cells in eight tubes, covering Vβ1–Vβ23 families. Staining of the Beta Mark TCRVβ Repertoire kit was performed according to the manufacturer’s instructions (Beckman Coulter, FR). Stained cells were captured using the Navios EX cytometer (Beckman Coulter). Cytobank software (*Beckman Coulter*) was used to analyze all available samples using the Navios Beckman Coulter cytometer. Data were compared with control samples and with mean percentages of expression of 24 TCR Vβ from a cohort of 85 normal specimens provided by the manufacturer [19]. All samples were analyzed using the same standardized antibody panel and flow cytometer, processed in sequential batches with identical instrument settings. Data quality was assessed by internal controls, adequate lymphocyte gating, and consistency of CD3+ cell recovery. No duplicate measurements were performed due to sample volume limitations.

### Statistical analysis

Data were analyzed using SPSS v20.0 (SPSS Inc., Chicago, IL, USA). Continuous variables were presented as medians (with 25th–75th percentiles) and categorical variables as frequencies and percentages. The Shapiro–Wilk test assessed normality. The Mann–Whitney *U* test was used for group comparisons. Nonparametric effect sizes were calculated using rank-biserial correlation for the Mann–Whitney *U* test. Associations were assessed using Spearman’s correlation, and effect sizes for associations are expressed as Spearman’s rho. A *p*-value < 0.05 was considered statistically significant.

## Results

### Characteristics of the study population

The study included 35 pediatric patients with CKD. The median age of the patients was 12.3 years, with a female-to-male ratio of 17:18. The most common underlying kidney disease was congenital anomalies of the kidney and urinary tract (CAKUT, *n* = 15), followed by ciliopathies (*n* = 7), glomerular diseases (*n* = 6), neurogenic bladder (*n* = 4), and tubulopathies (*n* = 2), while the etiology was unknown in one patient. The detailed clinical and laboratory characteristics of the CKD patients are summarized in the Suppl. Table.

There were no significant differences in age, sex distribution, or BMI-SDS adjusted for height between CKD patients and healthy controls (Table 1). However, the CKD patients had significantly lower height *z*-scores than the controls (*p* < 0.001). Within the CKD cohort, patients receiving dialysis exhibited significantly lower height and BMI *z*-scores compared to non-dialysis patients (*p* = 0.001 and *p* = 0.013, respectively).

**Table 1:** Demographic and anthropometric characteristics and lymphocyte counts and subsets of the study population

	CKD patients (*n* = 35**)**	Controls (*n* = 15)	*P*	Dialysis (*n* = 14)	Non-dialysis (*n* = 21)	*P*
Age, years	12.3 (8.6–19)	9.9 (7.7–15.2)	0.62	9.9 (8.4–17.8)	14.1 (9.2–19.9)	0.30
Sex, female/male, *n* (%)	17/18	7/8	0.55	9/5	8 /13	0.13
Height *z*-score	−1.1 (−2.1 to −0.4)	0.0 (−0.7 to 0.3)	** < 0.001**	124 (109–152)	148 (129–173)	** < 0.001**
BMI *z*-score for height age	0.07 (−1.10 to 1.9)	0.25 (−0.77 to 1.10)	0.24	−2.05 (−3.42 to −1.2)	−0.7 (−1.5–0.2)	** < 0.001**
eGFR* (mL/min/1.73 m^ 2^)	16.7 (8.4–30)	97.5 (93.25–117.05)	** < 0.001**	8.05 (5.82–11.52)	28.9 (18.6–39.05)	** < 0.001**
Total leukocytes (/μL)	7200 (6000–8800)	7000 (6300–7450)	0.94	7450 (5800–12025)	6800 (5950–7600)	0.198
Total lymphocytes (/μL)	2300 (1800–2900)	2500 (2150–3400)	0.25	2150 (1775–4275)	2300 (1750–2650)	0.78
CD3+ T-cells (/μL)	1603 (1239–2093)	1628 (1483–2218)	0.62	1694 (1231–2936)	1602 (1246–1888)	0.61
CD4+ T-cells (/μL)	984 (680–1238)	882 (714–1429)	0.94	1216 (720–1784)	949 (662–1079)	0.24
CD8+ T-cells (/μL)	685 (569–909)	629 (480–803	0.41	629 (486–801)	590 (357–997)	0.61
CD4/CD8 ratio	1.57 (1.23–2.03)	1.48 (1.25–1.83)	0.65	1.88 (1.46–2.46)	1.38 (1.14–1.74)	**0.013**
CD19+ B cells (/μL)	227 (158–379)	332 (202–466)	0.17	227 (171–323)	252 (140–687)	0.75
CD16/56 NK cells (/μL)	229 (122–329)	229 (122–329)	0.72	203 (97–429)	228 (153–335)	0.61
TCR α/β percentage	87.0 (83.4–89.4)	83.4 (82.7–86.0)	**0.026**	87.0 (82.4–90.1)	87.0 (82.9–89.3)	0.91
TCR γ/δ percentage	5.0 (3.8–7.0)	6.30 (4.50–6.80)	0.31	4.6 (3.0–5.2)	5.1 (4.1–7.2)	0.21
IgG (mg/dL)	903 (745–1110)	1004 (878–1089)	0.17	862 (724–1110)	902 (758–1109)	0.21
IgA (mg/dL)	115 (79–173)	109 (85–121)	0.94	141 (87–187)	110 (63–141)	0.86
IgM (mg/dL)	60 (51–77)	90 (76–108)	**0.002**	61 (40–76)	60 (54–77)	0.23

Data presented as median (25th and 75th percentiles). The Mann–Whitney *U* test was used for group comparisons.Values in bold indicate statistically significant differences (p <0.05 )

*CKD* chronic kidney disease, *BMI* body mass index, *NK* natural Killer, *TCR* T-cell receptor, *Ig* immunoglobulin, *eGFR* estimated glomerular filtration rate (*calculated using CKID-U25 equation)

### Lymphocyte profiles

Table 1 also presents the leukocyte and lymphocyte counts, as well as the subsets of the study population. Total leukocyte or lymphocyte counts did not differ between CKD patients and healthy controls, even though two patients had lymphocyte counts below 1500/µL. Four patients had low CD3+ counts, five had low CD4+, two had low CD8+, and four had reduced NK cells; however, group comparisons revealed no significant differences in CD3, CD4, CD8 T-cells, CD19 B cells, or NK cells. Notably, the proportion of TCR α/β was significantly higher in children with CKD than in controls (*p* = 0.026).

There were no differences in leukocyte or lymphocyte counts between dialysis and non-dialysis CKD patients, but the CD4/CD8 ratio was significantly higher in dialysis patients (*p* = 0.013), without any identifiable clinical or laboratory correlations.

### TCR Vβ repertoire

**Fig. 2:**
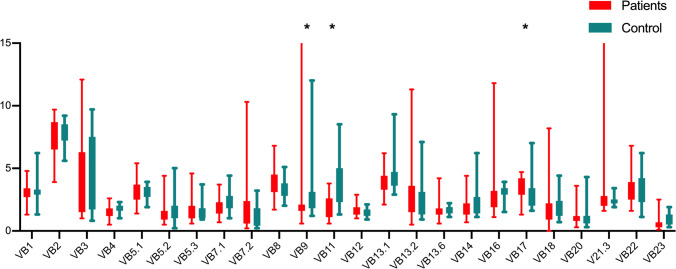
Comparisons of T-cell receptor (TCR) Vβ repertoire between CKD patients and controls. Only subsets with significant differences are shown. Since the repertoire of TCR Vβ was not a normal distribution, logarithmic transformation was used in the graph. Raw data are shown in Table 2. Bars represent median values with interquartile ranges (25th–75th percentiles) for each Vβ family. Statistically significant differences between groups are indicated with an asterisk (*p* < 0.05)

**Fig. 3:**
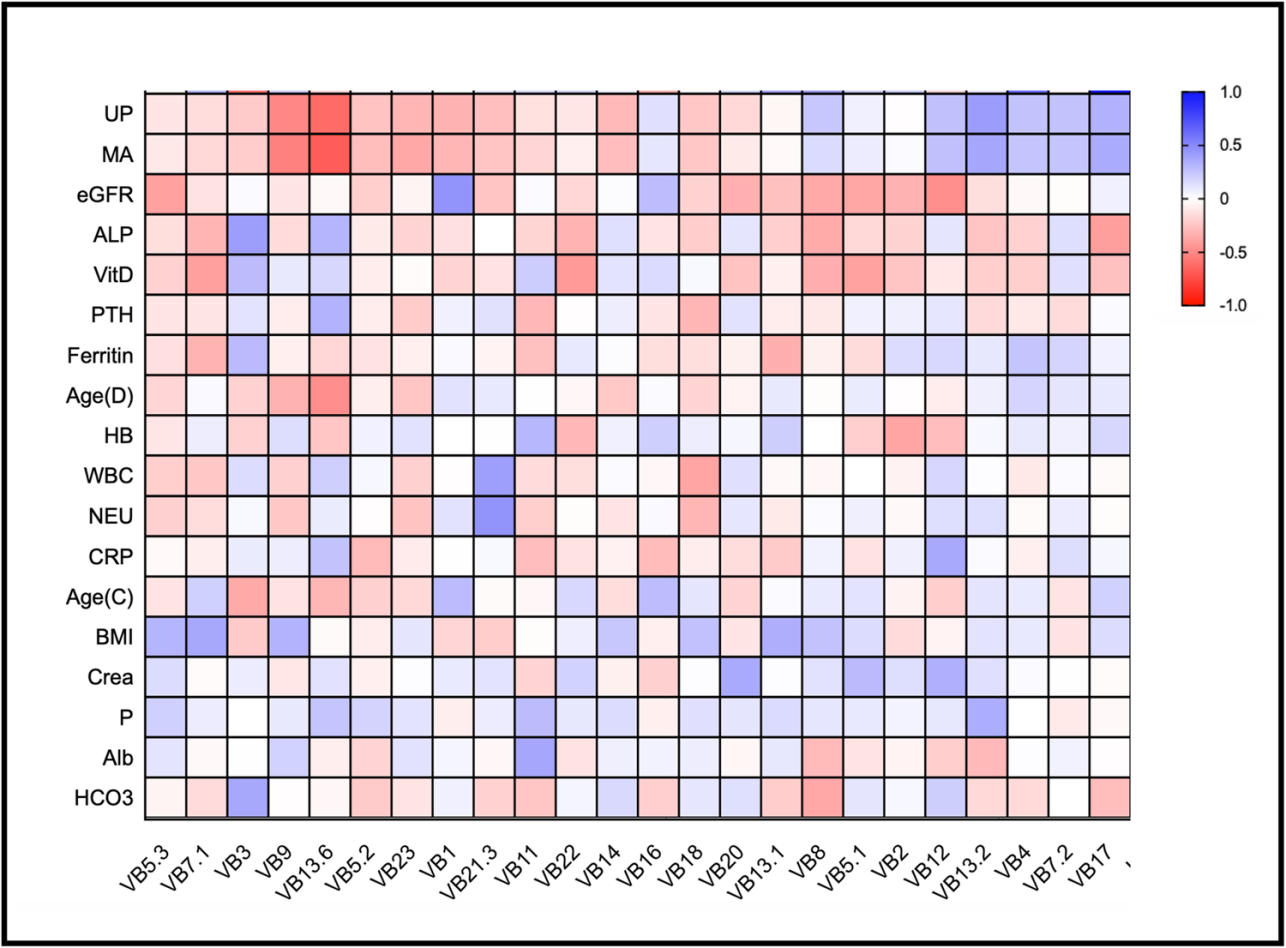
Variables are ordered based on hierarchical clustering to highlight correlated TCR Vβ subfamilies and clinical markers. *UP*, urinary proteinuria; *MA*, microalbuminuria; *eGFR*, estimated glomerular filtration rate; *ALP*, alkaline phosphatase; *VitD*, vitamin D; *PTH*, parathyroid hormone; *Age (D)*, age at diagnosis; *HB*, hemoglobin; *WBC*, white blood cell count; *NEU*, neutrophil count; *CRP*, C-reactive protein; *Age (C)*, chronological age; *BMI*, body mass index; *Crea*, serum creatinine; *P*, phosphorus; *Alb*, albumin; *HCO3*, bicarbonate; *TCR Vβ*, T-cell receptor variable beta family

CKD patients exhibited altered TCR Vβ distribution, characterized by significant reductions in TCR Vβ9 and Vβ11 expression and an increase in Vβ17 (p = 0.041, p = 0.001, and p = 0.014, respectively) (Fig. 2). In subgroup analysis, dialysis patients showed significantly lower Vβ11 and higher Vβ12 expression compared to non-dialysis patients (p = 0.034 and p = 0.048, respectively). Table 2 presents comparisons of the TCR Vβ repertoire between CKD patients and controls, and between dialysis and non-dialysis patients. Figure 3 presents the correlations between TCR Vβ subfamilies and clinical or biochemical parameters. Among the subfamilies that showed significant differences between CKD patients and healthy controls, lower Vβ9 expression correlated with lower BMI (*r* = 0.339, *p* = 0.047) and higher proteinuria (*r* = −0.500, *p* = 0.025). Reduced Vβ11 levels were associated with lower serum albumin (*r* = 0.378, *p* = 0.025), whereas increased Vβ17 was significantly associated with lower ALP levels (*r* = −0.410, *p* = 0.014). Additionally, elevated Vβ12, which was significantly higher in dialysis patients, showed positive correlations with CRP (*r* = 0.366, *p* = 0.030) and serum creatinine (*r* = 0.340, *p* = 0.046).

**Table 2:** TCR Vβ subsets of the study population

	CKD patients (*n* = 35)	Controls (*n* = 15)	*P*	Effect size	Dialysis (*n* = 14)	Non-dialysis (*n* = 21)	*P*	Effect size
TCR Vβ 1	3.0 (2.7–3.4)	3.0 (2.9–3.3)	0.99	0.01	3.15 (2.8–3.5)	3.0 (2.5–3.4)	0.31	0.56
TCR Vβ 2	7.6 (6.5–8.7)	7.7 (7.2–8.5)	0.79	0.05	7.9 (6.4–9.0)	7.5 (6.55–8.15)	0.45	0.53
TCR Vβ 3	4.4 (1.5–6.3)	5.2 (1.7–7.5)	0.57	0.10	5.5 (1.8–8.4)	4.2 (1.35–5.85)	0.135	0.61
TCR Vβ 4	1.6 (1.2–1.8)	1.7 (1.6–2.0)	0.21	0.22	1.65 (1.2–1.8)	1.6 (1.2–1.9)	0.83	0.46
TCR Vβ 5.1	3.1 (2.5–3.7)	2.9 (2.7–3.5)	0.98	0.01	3.3 (2.45–4.3)	2.9(2.35–3.55)	0.28	0.57
TCR Vβ 5.2	1.2 (0.9–1.6)	1.6 (1.0–2.0)	0.19	0.24	1.1 (0.8–1.75)	1.2 (1.0–1.5)	0.70	0.48
TCR Vβ 5.3	1.4 (1.0–2.0)	1.4 (1.0–1.8)	0.98	0.01	1.3 (0.95–2.1)	1.4 (1.0–1.95)	0.88	0.46
TCR Vβ 7.1	1.8 (1.4–2.3)	2.4 (1.75–2.8)	0.12	0.28	1.75 (1.6–2.25)	2.0 (1.4–2.3)	0.73	0.48
TCR Vβ 7.2	1.8 (0.6–2.4)	1.2 (0.4–1.8)	0.18	0.24	1.65 (0.55–2.45)	1.7 (0.55–2.3)	0.91	0.45
TCR Vβ 8	3.7 (3.1–4.5)	3.6 (2.8–3.8)	0.35	0.17	3.75 (3.1–4.6)	3.6 (3.15–4.45)	0.829	0.47
TCR Vβ 9	1.8 (1.6–2.1)	2.4 (1.8–3.1)	**0.041**	0.37	1.75 (1.4–2.2)	1.9 (1.65–2.25)	0.56	0.51
TCR Vβ 11	1.9 (1.1–2.6)	3.1 (2.3–5.0)	**0.001**	0.61	1.2 (0.9–2.2)	2.0 (1.3–2.75)	**0.034**	0.68
TCR Vβ 12	1.6 (1.3–1.9)	1.5 (1.2–1.7)	0.21	0.23	1.8 (1.5–2.1)	1.4 (1.3–1.8)	**0.048**	0.66
TCR Vβ 13.1	3.7 (3.3–4.4)	3.9 (3.6–4.7)	0.22	0.22	3.5 (3.0–4.1)	4.0 (3.3–4.6)	0.45	0.53
TCR Vβ 13.2	2.4 (1.5–3.6)	2.3 (1.25–3.1)	0.68	0.07	2.2 (1.15–4.25)	2.4 (1.6–3.3)	0.91	0.45
TCR Vβ 13.6	1.6 (1.3–1.8)	1.7 (1.4–1.9)	0.49	0.12	1.6 (1.3–2.1)	1.5 (1.25–1.8)	0.31	0.56
TCR Vβ 14	1.9 (1.3–2.2)	2.1 (1.4–2.7)	0.18	0.24	1.85 (1.7–2.1)	1.9 (1.25–2.25)	0.78	0.47
TCR Vβ 16	2.7 (1.9–3.2)	3.1 (2.9–3.4)	0.054	0.35	2.2 (2.0–2.9)	2.8 (1.85–3.55)	0.28	0.56
TCR Vβ 17	3.5 (2.9–4.2)	2.3 (2.0–3.4)	**0.014**	0.44	3.55 (2.8–3.97)	3.5 (2.9–4.4)	0.65	0.49
TCR Vβ 18	1.5 (0.9–2.2)	1.4 (1.2–2.4)	0.36	0.16	1.7 (0.8–2.35)	1.4 (1.0–2.1)	0.855	0.46
TCR Vβ 20	0.9 (0.8–1.2)	0.8 (0.6–1.2)	0.25	0.21	1.05 (0.8–1.3)	0.9 (0.7–1.15)	0.29	0.56
TCR Vβ 21.3	2.2 (2.0–2.8)	2.3 (2.2–2.5)	0.62	0.09	2.3 (20–2.5)	2.2 (1.9–2.85)	0.83	0.47
TCR Vβ 22	3.3 (2.5–3.9)	3.3 (2.3–4.2)	0.88	0.03	3.5 (2.8–4.6)	3.1 (2.3–3.85)	0.26	0.57
TCR Vβ 23	0.5 (0.3–0.7)	0.7 (0.5–1.3)	0.06	0.34	0.5 (0.3–0.9)	0.5 (0.4–0.7)	0.93	0.45

Data presented as median (25th and 75th percentiles). Effect sizes are reported as rank-biserial correlation (Mann–Whitney *U*). Values in bold indicate statistically significant differences (p  <0.05). *TCR* T-cell receptor

## Discussion

This study provides additional evidence of immune dysregulation in pediatric CKD, characterized by selective expansion and depletion of specific TCR Vβ subsets, consistent with premature immune senescence. These changes reflect selective, non-uniform remodeling of the T-cell repertoire diversity rather than a simple quantitative reduction in lymphocytes. The observed repertoire restriction in this pediatric population supports the hypothesis that immune aging in CKD may begin early in life and may be driven by uremic and proinflammatory milieu.

Although premature immune senescence has been previously suggested in pediatric CKD populations, particularly in transplant recipients or immunosuppressed cohorts, our study provides the first detailed characterization of TCR Vβ repertoire skewing in non-immunosuppressed children with advanced native-kidney CKD [11, 16]. By excluding patients receiving immunosuppressive therapy, our findings demonstrate that qualitative TCR repertoire remodeling occurs independently of transplantation-related factors, supporting a direct effect of CKD-associated uremia and inflammation on immune aging.

The pediatric setting of this study provides a unique perspective on immune alterations in CKD. Unlike adult cohorts previously examined for TCR repertoire changes in CKD [15, 16], our patients predominantly had CAKUT and no comorbidities such as type 2 diabetes or congestive heart failure, which independently accelerate immune aging. The cohort’s median age of 12 years minimizes long-term influences of metabolic, environmental, and pharmacological factors that usually affect immune profiles in adults [1, 9, 16, 20]. Nevertheless, children with CKD exhibited a distinct pattern of TCR Vβ skewing, characterized by reduced expression of Vβ9 and Vβ11 and increased usage of Vβ17. Further disruption was observed in dialysis patients, which was marked by lower Vβ11 and higher Vβ12 levels, as also observed in adult hemodialysis populations [15, 16]. These findings suggest that CKD itself may trigger early immune senescence, long before the classical features of aging become evident.

Our results support that uremia and chronic inflammation may act as major contributors for T-cell repertoire shift in pediatric CKD. Evidence from adult studies describes similar processes in which chronic exposure to uremic toxins, oxidative stress, and persistent antigenic stimulation promote T-cell exhaustion and clonal restriction, the so-called “inflammaging” phenotype [9, 16, 20, 21]. In this context, the observed increase in Vβ12 expression in association with higher CRP and creatinine levels should be interpreted cautiously and viewed as reflecting an inflammatory and uremic milieu rather than a direct causal relationship [2, 16, 21]. In parallel, the associations of reduced Vβ9 with low BMI and proteinuria, and reduced Vβ11 with hypoalbuminemia, indicate that malnutrition and protein loss may exacerbate inflammatory and metabolic stress, thereby accelerating repertoire restriction. Proteinuria may enhance immune activation, promoting chronic T-cell stimulation and oligoclonal restriction of specific Vβ subsets [15]. Hypoalbuminemia, as a marker of inflammation and nutritional deficiency, may contribute to disturbed T-cell homeostasis [21]. These observations suggest that chronic inflammation, uremic toxicity, and nutritional deficiency work together to alter the T-cell repertoire in children with CKD. Further mechanistic studies integrating functional immune assays, cytokine profiling, and uremic toxin analysis are required to elucidate these pathways.

Although the total lymphocyte count and the major subsets (CD3, CD4, CD8, CD19, NK) did not differ between CKD patients and healthy controls, CKD patients showed an increased TCR α/β percentage, and dialysis patients had an increased CD4/CD8 ratio, possibly indicating immune adaptation to chronic stress. This finding is consistent with previous reports suggesting that CKD-related immune dysregulation can occur even without classical lymphopenia [6]. Other studies have demonstrated that high CD4/CD8 ratios are associated with more severe disease activity, poorer kidney function, and a higher risk of progression to stage 5 CKD [22]. Meanwhile, altered T-cell counts in advanced CKD have been shown to increase susceptibility to infections and adverse outcomes [23]. Furthermore, reduced absolute numbers and percentages of γδ T-cells have also been reported in pediatric dialysis patients [24]. Together, these findings indicate that the immune dysfunction in CKD is best described as compositional (subset-specific) remodeling rather than lymphocyte depletion.

The strengths of this study include its novelty as the first pediatric investigation of the TCR Vβ repertoire using multiparameter flow cytometry, exclusion of patients on immunosuppressive therapy, and relatively long disease duration enabling assessment of chronic immune effects. In addition, our study suggests that specific TCRVβ shifts may be predictive of certain clinical entities like disease activity or progression.

Nonetheless, several limitations need to be acknowledged. First, the relatively small sample size, particularly in the dialysis and control subgroups, limited statistical power and precluded multivariable modeling to estimate adjusted effect sizes; therefore, the reported associations should be interpreted as exploratory and hypothesis-generating. Second, our immunophenotyping was limited to peripheral blood TCR Vβ repertoire distribution and did not include separate CD4+ and CD8+ subset analysis, naive/memory T-cell phenotyping, or functional immune assays and cytokine profiling, which would provide deeper mechanistic insight into immune senescence in CKD. Finally, the cross-sectional design and lack of longitudinal clinical outcome data, particularly regarding infection episodes and vaccine responses, limit conclusions about the direct assessment of the clinical implications of the observed TCR repertoire skewing, which should be addressed in future prospective studies.

## Conclusions

We find that senescence-like changes of the TCR Vβ repertoire in pediatric CKD occur without overt lymphopenia or relevant changes in lymphocyte subsets. The presence of increased Vβ12, as observed mainly in dialysis patients, and associated with higher CRP and creatinine levels, points toward inflammation- and uremia-related immune remodeling. These findings support the concept that immune aging in CKD may begin early in life and highlight the potential of TCR Vβ repertoire analysis as a research tool for identifying early immune senescence in pediatric CKD. However, based on the cross-sectional design and relatively limited sample size, the results need to be interpreted as hypothesis-generating, requiring further confirmation in larger, longitudinal studies.

## Supplementary Information

Below is the link to the electronic supplementary material.

ESM1 (PPTX 422 KB)

ESM2 (DOCX 15.1 KB)

## Data Availability

All data generated or analysed during this study are included in this published article and its supplementary information files.
