# Supplementary material for: T-cell receptor Vβ repertoire skewing reflects premature immune senescence in children with chronic kidney disease
Source: Pediatr Nephrol. 2026 Mar 25;41(9):2923–30. doi: 10.1007/s00467-026-07267-w (PMC13424352; doi:10.1007/s00467-026-07267-w)
Supplement: Supplementary file 1 — (PPTX 422 KB) [file 467_2026_7267_MOESM1_ESM.pptx]

## Slide 1
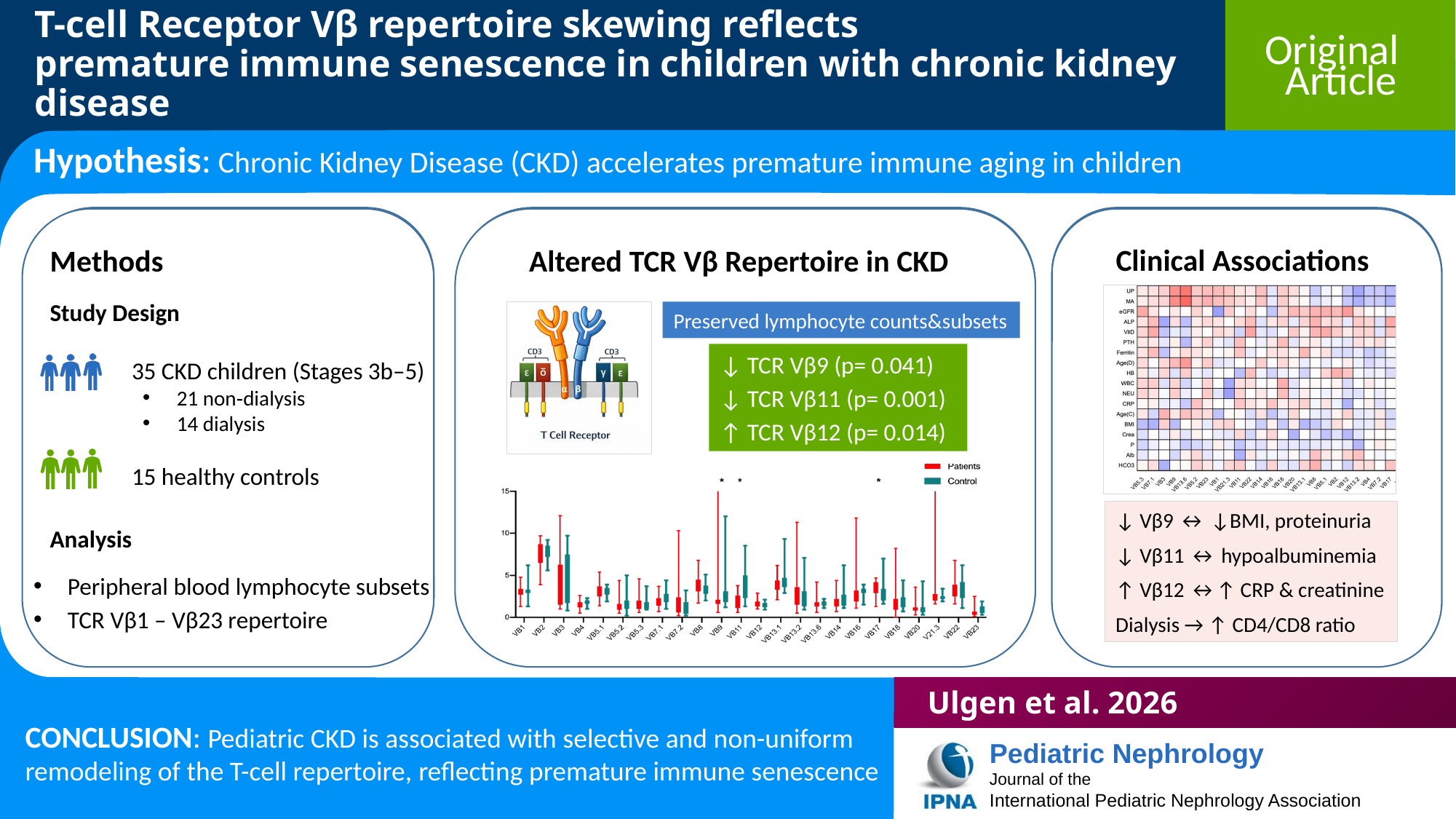

T-cell Receptor Vβ repertoire skewing reflects
premature immune senescence in children with chronic kidney disease
Hypothesis: Chronic Kidney Disease (CKD) accelerates premature immune aging in children
Clinical Associations
Altered TCR Vβ Repertoire in CKD
Methods
 Study Design
 35 CKD children (Stages 3b–5)
21 non‑dialysis
14 dialysis
        15 healthy controls
 Analysis
Peripheral blood lymphocyte subsets
TCR Vβ1 – Vβ23 repertoire
Preserved lymphocyte counts&subsets
↓ TCR Vβ9 (p= 0.041)
↓ TCR Vβ11 (p= 0.001)
↑ TCR Vβ12 (p= 0.014)
↓ Vβ9 ↔ ↓BMI, proteinuria
↓ Vβ11 ↔ hypoalbuminemia
↑ Vβ12 ↔↑ CRP & creatinine
Dialysis → ↑ CD4/CD8 ratio
Ulgen et al. 2026
CONCLUSION: Pediatric CKD is associated with selective and non-uniform remodeling of the T-cell repertoire, reflecting premature immune senescence
