# Supplementary material for: T-cell receptor Vβ repertoire skewing reflects premature immune senescence in children with chronic kidney disease
Source: Pediatr Nephrol. 2026 Mar 25;41(9):2923–30. doi: 10.1007/s00467-026-07267-w (PMC13424352; doi:10.1007/s00467-026-07267-w)
Supplement: Supplementary file 2 — (DOCX 15.1 KB) [file 467_2026_7267_MOESM2_ESM.docx]

## **Supplemental Table:** Clinical and laboratory characteristics of the patients

|  | **All CKD patients**  **(n = 35)** | **Dialysis patients**  **(n = 14)** | **Non-dialysis CKD patients**  **(n = 21)** | **p** |
| --- | --- | --- | --- | --- |
| **Clinical features** | | | |  |
| Sex, female, n (%) | 17 (49) | 9 (64) | 8 (38) | 0.13 |
| Age, years | 12.3 (8.6–19.0) | 10.0 (8.4-17.8) | 14.1 (9.2-20.0) | 0.31 |
| Age at diagnosis of primary kidney disease, years | 2.6 (0.01–8.3) | 0.5 (0.0–4.5) | 4.7 (0.3–9.7) | 0.15 |
| Duration of CKD, years | 4.9 (2.5–9.5) | 4.2 (1.6–8.7) | 5.8 (2.8–9.8) | 0.36 |
| Duration of CKD 5, years | — | 2.1 (1.1–5.9) | — | — |
| **Laboratory findings** | | | |  |
| Urea, mg/dL | 102 (68–124) | 122.5 (99.8–189) | 93.0 (54–108.5) | **0.004** |
| Creatinine, mg/dL | 3.46 (1.8–5.3) | 5.65 (4.25–8.4) | 2.34 (1.6–3.4) | **<0.001** |
| Calcium, mg/dL | 9.3 (8.7–9.6) | 9.1 (8.6–9.4) | 9.9 (9.5–10.1) | 0.11 |
| Phosphorus, mg/dL | 4.5 (3.7–5.1) | 4.6 (3.5–6.1) | 4.5 (3.8–4.9) | 0.75 |
| Alkaline phosphatase, IU/L | 176 (110–275) | 199 (124–374) | 159 (103–272) | 0.29 |
| 25 (OH) Vitamin D, ng/mL | 19 (12.6–26) | 14.7 (7.8–29.9) | 21 (13–25.8) | 0.21 |
| Parathyroid hormone, pg/mL | 108 (58.5–299) | 266 (166–679) | 79.4 (44.4–185) | **0.008** |
| C-reactive protein, mg/L | 0.71 (0.41–2.7) | 0.9 (0.6–2.6) | 0.7 (0.4–3.05) | 0.63 |

CKD; chronic kidney disease

Data are presented as median (25th–75th percentile)
